# All-optical control of charge-trapping defects in rare-earth doped oxides

**DOI:** 10.1515/nanoph-2024-0635

**Published:** 2025-02-14

**Authors:** Leonardo V. S. França, Shaan Doshi, Haitao Zhang, Tian Zhong

**Affiliations:** Pritzker School of Molecular Engineering, 2462 The University of Chicago, Chicago, IL 60637, USA; Corning Research & Development Corporation, Sullivan Park, Painted Post, New York, 14870, USA

**Keywords:** charge trapping, defects, optical storage, rare-earth ions, yttrium oxide

## Abstract

Charge-trapping defects in crystalline solids play important roles in applications ranging from microelectronics, optical storage, sensing and quantum technologies. On one hand, depleting trapped charges in the host matrix reduces charge noise and enhances coherence of solid-state quantum emitters. On the other hand, stable charge traps can enable high-density optical storage systems. Here we report all-optical control of charge-trapping defects *via* optical charge trapping (OCT) spectroscopy of a rare-earth ion doped oxide (Y_2_O_3_). Charge trapping is realized by low intensity optical excitation in the 200–375 nm range. Charge detrapping or depletion is carried out by optically stimulated luminescence (OSL) under 532 nm stimulation. Using a Pr-doped Y_2_O_3_ polycrystalline ceramic host matrix, we observe charging pathways *via* the inter-band optical absorption of Y_2_O_3_ and *via* the 4f-5d transitions of Pr^3+^. We demonstrate effective control of the density of trapped charges within the Y_2_O_3_ matrix at ambient environment. These results point to a viable method for controlling the local charge environment in rare-earth doped crystals *via* all-optical means, and pave the way for further development of efficient optical storage technologies with ultrahigh storage capacity, as well as for the localized control of quantum coherence in rare-earth doped solids.

## 1 Introduction

Rare-earth ion doped crystals are used in wide-ranging applications including lasers, optoelectronics, displays, scintillation, optical storage and quantum information science. One of the most common host crystals is metal oxides, where rare-earth dopants can substitute constituent atoms in the host as color centers. Lattice defects such as oxygen vacancies and interstitial oxygens are commonly present in these oxides [1], [2], [3], and the density of which critically depends on the synthesis method, growth conditions and post-growth treatment [4], [5], [6]. These defects promote new energy levels within the band-gap of the host matrix, thus forming electron or hole traps with stable charge state [7], [8], [9]. For quantum information applications, where the rare-earth ions act as memories or quantum emitters, any charged defect proximal to the rare-earth dopants could significantly alter their local charge environment and impact the quantum coherence properties of the rare-earth ions. A notable effect is the optical spectral diffusion [10] in which the optical linewidth of a rare-earth ion is broadened due to the coupling to the fluctuating charge states of nearby traps [11]. Such effects can be particularly pronounced when rare-earth ions are embedded in nanophotonic structures such as waveguides and cavities, in which dynamic charge noise on the surfaces degrades ions’ coherence properties [12], [13]. This motivates the investigation of techniques to control the charge state of these trap defects, which can mitigate local charge noise in the host matrix, hence enhancing the optical coherence times of rare-earth dopants. Furthermore, deterministic control of charge traps can also enable optical storage technologies with high density storage capacity [14], [15], [16], [17].

In this article, we report an experimental technique for all-optical control of charge-trapping defects, i.e., optical charge trapping (OCT) spectroscopy. The OCT experiment consists of two steps. It begins with charging the sample with optical illumination at a certain energy. Subsequently, the density of trapped charges is measured by means of optically stimulated luminescence (OSL), which is the radiative recombination during optical stimulation after charge trapping. Using OCT spectroscopy, one can correlate the density of trapped charges with the preceding optical excitation energy (i.e., charging energy). It is worth noting that OSL itself is a standard technique in luminescence dosimetry [18], [19], [20], and it has also shown to be useful in the exploration of persistent luminescence phosphors and optical storage systems [21], [22], [23]. Here, OSL is used as a charge readout technique in OCT to reveal the optical charge trapping process.

Using a praseodymium (Pr^3+^) doped yttrium oxide (Y_2_O_3_) host matrix, we observed that charging by optical means can be realized *via* two main pathways: first, inter-band transition under UV excitation at 215 nm; second, *via* the 4f-5d optical transitions (275 nm) of the Pr^3+^ dopants. Both charging processes are efficient, requiring optical excitation intensity as low as ∼5 μW/cm^2^. Exploiting this, we demonstrated low-power all optical charge control and on-demand readout in Pr:Y_2_O_3_. These results provide a pathway for effectively controlling the charge environment in rare-earth doped crystals, and paves the way for future development of efficient high-density optical storage technology, as well as for the localized control of quantum coherence in rare-earth- doped solids.

## 2 Materials and methods

**Figure 1:**
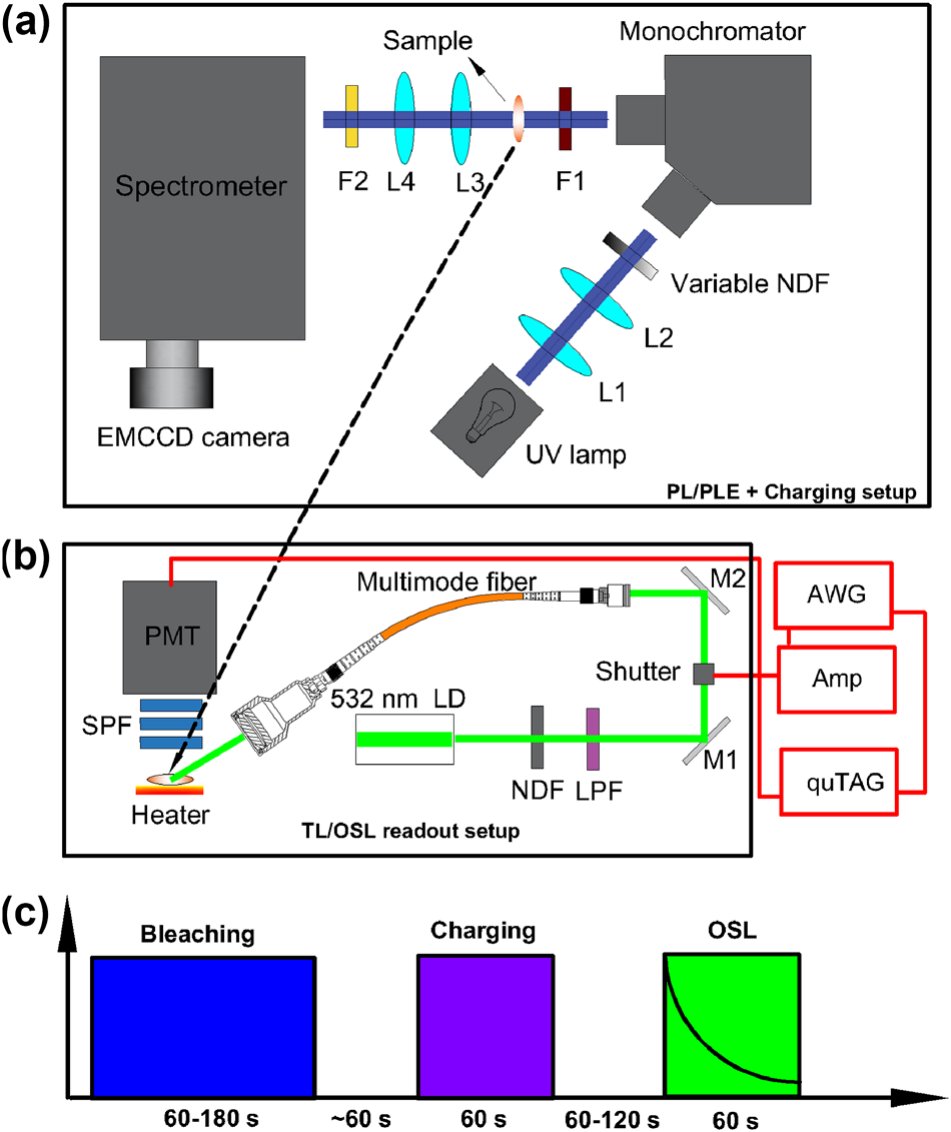
Schematic setup for the PL/PLE spectroscopy and TL/OSL emission readout, as well as the time sequence of the OCT measurement. (a) Setup for the PL/PLE and charging experiments. (b) Experimental setup for the TL/OSL intensity readout. To collect OSL emission spectra, the optical fiber delivering the stimulation laser was placed in parallel to the initial UV beam and facing the sample (top panel, not shown). L1–L4: lenses; NDF: neutral density filter; F1: excitation filter; F2: detection filter; LPF: long pass filter; SPF: short pass filter; LD: laser diode; M1, M2: mirrors; AWG: arbitrary wave generator; Amp: high voltage amplifier; quTAG: a photon counter. For the TL measurements, one bandpass filter and two optical windows were placed in front of the PMT, instead of three shortpass filters (details in the text). (c) Time-sequence of the events to acquire the OCT spectrum. There is a wait time between bleaching and charging, as well as between charging and OSL readout. During the wait times, sample is moved between different setups.

We carried out this study using both nominally undoped and 20 parts per million (ppm) Praseodymium (Pr) doped Y_2_O_3_ polycrystalline ceramics. The size of the undoped sample is 5 × 4 × 0.4 mm^3^ and the Pr-doped sample has a 13 mm diameter with 0.9 mm thickness. The synthesis method for these samples was described in detail in Ref. [24]. Photoluminescence (PL) and photoluminescence excitation (PLE) spectra were collected with a high-resolution spectrometer (SpectraPro HRS-750, Princeton Instruments) with a 1,800 g/mm groove density grating blazed at 500 nm coupled to an EMCCD camera (Pylon 400BRX, Teledyne). For excitation, as depicted in Figure 1a, a deuterium light source with 15 W/m^2^ intensity (SL-3, StellarNet) was used, and the broadband source spectrum was filtered by a 100 mm UV monochromator (H10-61, Jobin Yvon Horiba) with a grating blazed at 250 nm. Appropriate filters were inserted in the excitation and the detection paths. The PL spectra were corrected for the wavelength dependent system responsivity. Both PL and PLE spectra were background subtracted. All spectra were collected with the EMCCD camera kept at −120 °C.

In order to acquire the OCT spectrum, charge trapping was carried out with the same UV source and monochromator described previously. A variable neutral density filter was used to adjust the charging optical power, which was monitored with a power sensor (S120VC, Thorlabs) placed at sample position. Charging spot size at the sample position was about 3 mm diameter. After charging, luminescence readout was carried out during optical stimulation. The experimental schematics for the OSL intensity readout is shown in Figure 1b. A 532 nm diode laser was used for continuous wave optical stimulation and was attenuated to an intensity of 5 mW/cm^2^ to reduce backscattered light. Spot size at the sample position was about 7 mm in diameter. Light detection was performed by a photomultiplier tube (PMT) (230–700 nm range, H10682-110 model, Hamamatsu) with three 500 nm shortpass filters. The OSL signal was recorded by a photon counter (quTAG from qutools) over 60 s with a 200 ms integration time. An optical shutter and an arbitrary wave generator were integrated for synchronizing the data acquisition with optical stimulation. Charge bleaching was performed prior to all OSL measurements to ensure depletion of residual trapped charges, which was done by sustained illumination at 532 nm for 60 s. If the background did not reach our reference values, i.e., approximately 300 counts per second (when stimulation is on), then bleaching was completed by a 470 nm flashlight (300 mW maximum optical power). Total bleaching times were between 60 s and 180 s. Figure 1c represents a time sequence of the events to collect the OCT spectrum: first, bleaching for residual charge depletion; second, charging *via* UV excitation and third, OSL intensity readout.

OSL emission spectra were collected with the same detection setup used for the PL/PLE measurements. Using the same 532 nm optical stimulation, the OSL emission spectra were collected over the 350–710 nm wavelength range, and were accumulated over 3 s of optical stimulation with 200 ms integration time. The same bleaching procedure described previously took place before each spectrum acquisition. The OSL emission spectra were corrected for the wavelength dependent system responsivity.

Alternatively, thermal stimulation of the trapped charges can be performed, giving rise to thermoluminescence (TL) [25], [26]. TL measurements were performed with a setup that comprises a custom-made heating system, i.e., a ceramic heater with a PID temperature controller, and a PMT (300–650 nm range, H11870-01 model, Hamamatsu) connected to the photon counter. Light was filtered out by a 450 nm band pass filter (40 nm linewidth) and two 5 mm fused quartz optical windows were placed between filter and heater to prevent thermal degradation of the PMT. Signal from the PMT was processed by the photon counter, which was synchronized with the temperature controller. The heating rate used during TL measurements was 1 °C/s and the PMT response was integrated over 1 s. Figure 1b shows the setup for the TL measurement.

## 3 Results and discussion

Pr-doped Y_2_O_3_ crystals have been previously studied [27], [28], [29]. Praseodymium ions substitute yttrium ions in the lattice and that is expected since their trivalent charged states have similar ionic radii (0.90–0.99 Å) for the same coordination number (6). The 4f-4f transitions of Pr^3+^ in the visible range are within 450–500 nm (^3^H_4_ → ^3^P_*J*_) and 580–750 nm (^1^D_2_ → ^3^H_4_) ranges. 4f-5d transitions lie in the 250–360 nm range and the two different substitutional sites C_2_ and S_6_ correspond to different absorption bands (280 nm and 317 nm center emissions) [28]. 4f-4f transitions in the near infrared are also possible but our study is limited to UV and visible ranges.

**Figure 2:**
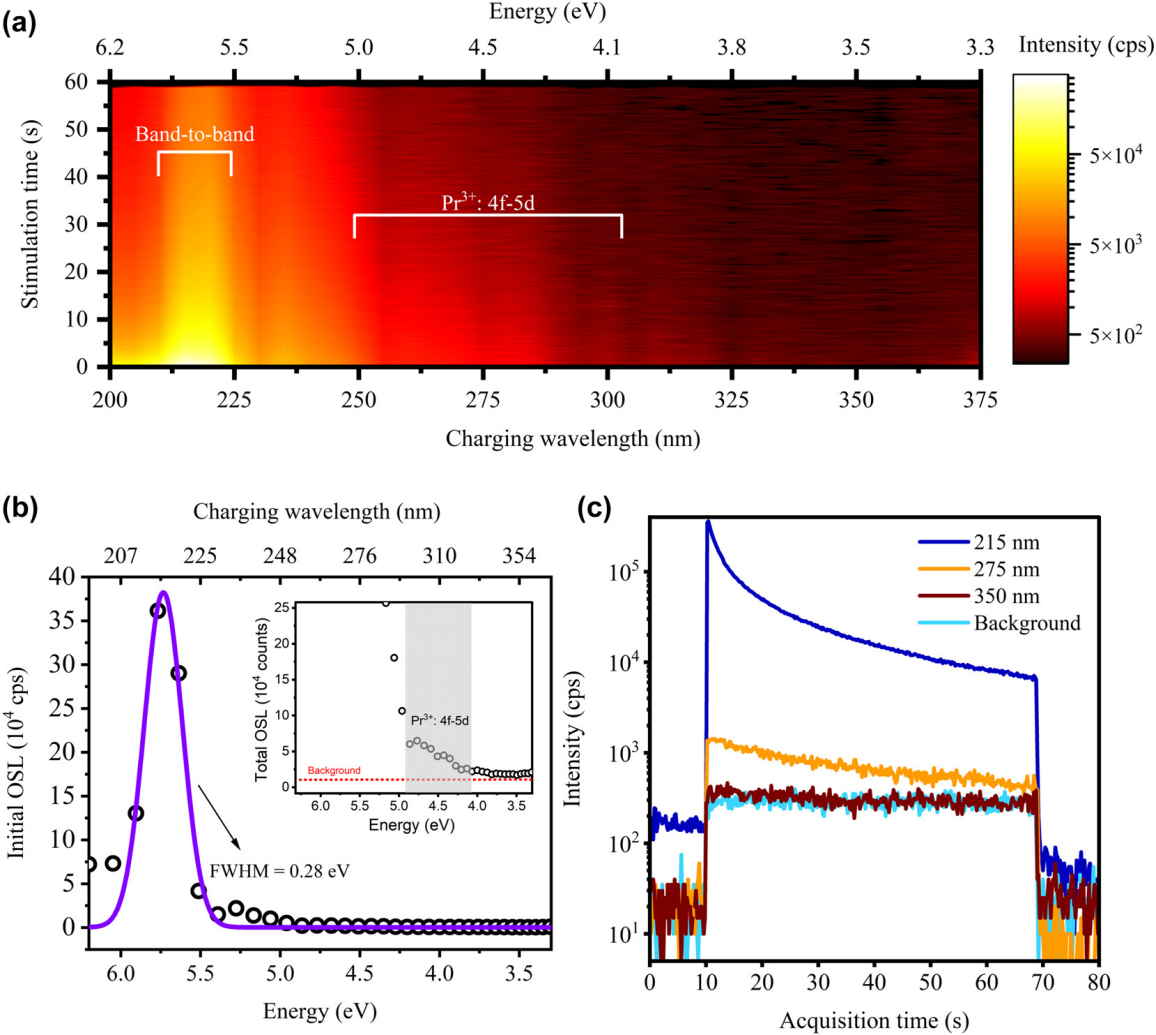
OCT spectrum and OSL decay curves of the Pr:Y_2_O_3_ sample. (a) OCT spectrum plotted as a heatmap. Charging optical power used was 300 nW over 60 s. Step size and charging linewidths: 5 nm. (b) Initial OSL intensity plotted against charging wavelengths. Initial OSL intensity corresponds to the first 200 ms stimulation cross-section of Figure 2a. Scatter points represent experimental data and solid lines represent a single peak Gaussian fit. Inset shows a zoomed-in region of the integrated OSL over 60 s of stimulation. (c) OSL decay curves after different charging wavelengths. CW stimulation started 10 s after starting data acquisition and was shut down after 60 s of stimulation. Background represents the PMT response after full depletion of residual trapped charges.

OSL measurements were performed after charging the sample with different excitation wavelengths in the 200–375 nm range with a 5 nm step. Figure 2a shows the OCT spectrum. We observed most pronounced charging effect at around 215 nm and clear OSL decays are also observed at wavelengths towards 300 nm. Charging in the 300–375 nm range did not yield significant OSL. Charging under 215 nm excitation is expected since it corresponds to the inter-band transition of Y_2_O_3_, with experimental band-gap of 5.8–6.0 eV [30], [31], [32]. It is worth to mention that here the OCT spectrum should not be confused with the OSL stimulation spectrum [33]. The former informs possible optical pathways that lead to charge trapping. The latter is related to a particular charge trap and provides information about the trap photoionization cross-section dependence on the stimulation wavelengths [34], [35], [36].

**Figure 3:**
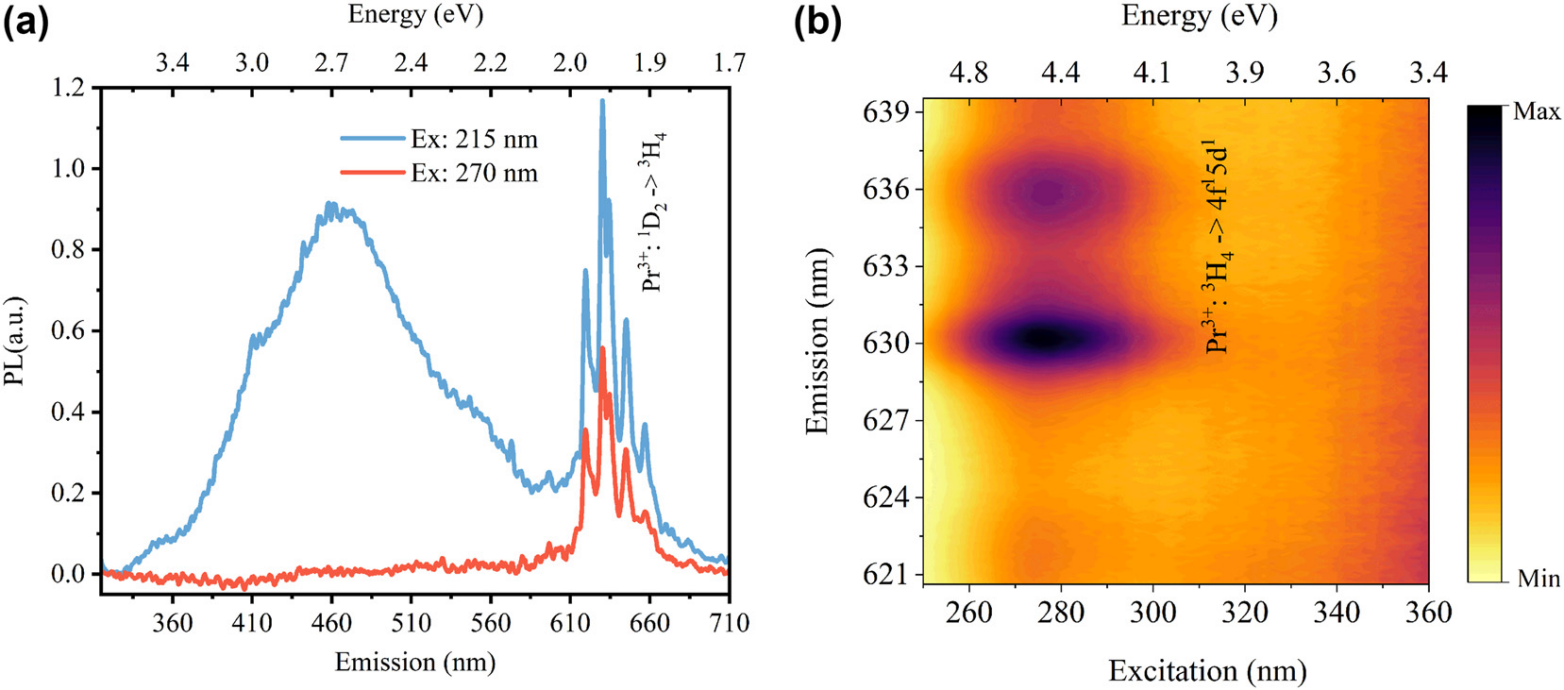
PL and PLE measurements. (a) Photoluminescence (PL) spectra under 215 nm and 270 nm excitations. KG3 Schott glass filter, 315–710 nm transmission (FGS900 Thorlabs) was used. (b) Excitation-emission spectrum with grating center wavelength set as 630 nm. A 640 nm bandpass filter (75 nm bandwidth, 67036 Edmund optics) was used before detection. The emission lines appear broadened because the spectrometer slit was enlarged to improve light collection.

**Figure 4:**
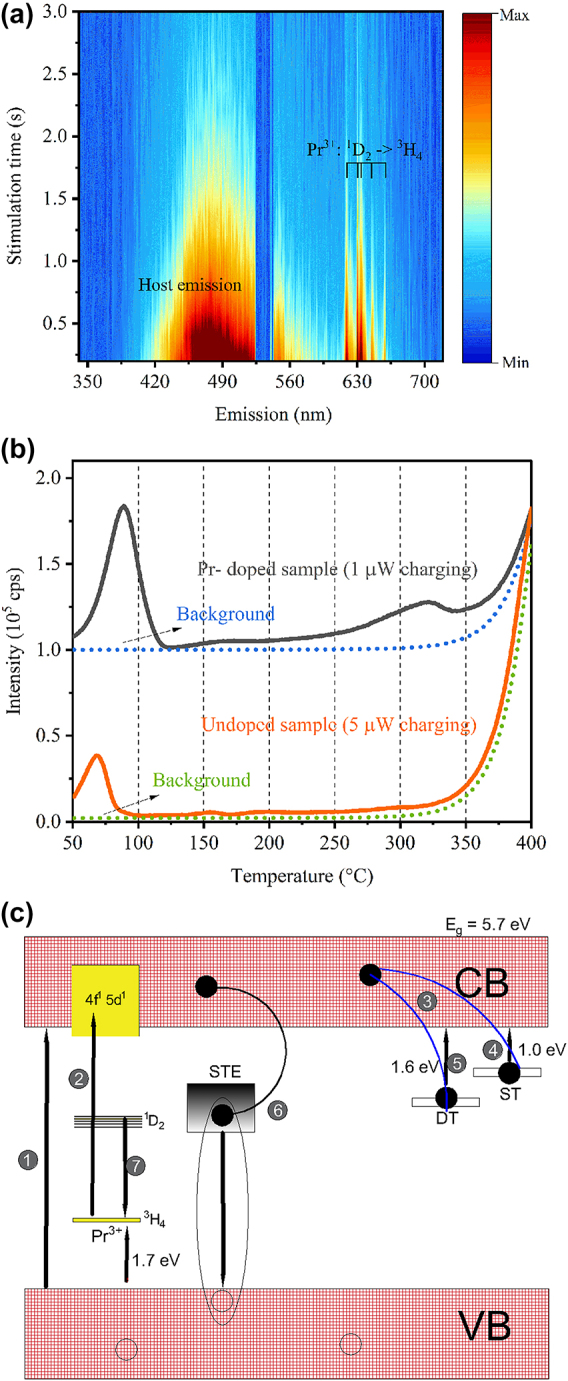
OSL emission spectra, TL measurements and a charge-recombination model. (a) OSL emission spectra after 215 nm charging (10 nm linewidth, 1 μW over 60 s). The discontinuity around 530 nm arises from the 532 nm notch filter placed before detection. (b) Thermoluminescence (TL) curves and the background (readout right after TL) for both Pr-doped and undoped Y_2_O_3_ samples. Charging was performed over 60 s exposure. (c) Charging-recombination model for Pr:Y_2_O_3_. 1 represents the band-to-band excitation; 2 represents 4f-4f5d transition of Pr^3+^; 3 represents charge trapping processes; 4 represents spontaneous release of charges at room temperature; 5 represents charge detrapping during optical stimulation; 6 represents radiative recombination *via* self-trapped exciton relaxation and 7 represents radiative recombination *via* 4f-4f transition of Pr^3+^. Non-radiative relaxation to ^1^D_2_ is not shown here. Solid black circles and empty circles represent electrons and holes, respectively.

Figure 2b plots the initial OSL intensity against the charging wavelengths. The peak centered at 5.7 eV is fitted to a Gaussian function, yielding a spectral width of 0.3 eV. The intensity on the left side of 5.7 eV is higher than the right side. This is expected because energies higher than the band-gap are more effective at electron-hole pair generation. Here, the measurement error is 3 % estimated from the standard deviation of five measurements (Figure S1, Supplementary Materials). Excitations between 4.1 eV (302 nm) and 4.8 eV (258 nm) can also charge the sample, as shown in the inset of Figure 2b. This spectral band matches with the ^3^H_4_ → 4f-5d transition of Pr^3+^, as verified by the PLE spectrum shown later in Figure 3b. This charging pathway, though yielding lower OSL intensity, is actually highly efficient, considering the Pr dopant concentration of only 20 ppm in Y_2_O_3_. This leads to an interesting prospect of using the 4f-5d optical transition of individual Pr dopants to efficiently charge nearby traps in the host. In addition, the undoped sample did not exhibit charging under excitation at the 4f-5d transitions of Pr^3+^ (Figure S2, Supplementary Materials). It is worth mentioning that the OSL response of the undoped sample after 215 nm charging was much lower than that of the Pr-doped sample, by a factor of 50. This could be due to an increased defect density during Pr doping, as corroborated by the thermoluminescence results (see Figure 4b).

Figure 2c shows the OSL signal as a function of the stimulation time after different charging wavelengths (cross sections from the OCT spectrum). OSL following 215 nm charging exhibited the highest intensity, as expected. After 60 s of stimulation time, the OSL intensity was at least 10 times the background, which is the PMT response from a completely bleached sample. Spontaneous emission was also observed after 215 nm charging as indicated by the offset increase before starting the stimulation (see also Figure S3, Supplementary Materials). These results suggest that the Pr:Y_2_O_3_ sample consists of at least two trapping centers, a shallow one which is responsible for the spontaneous emission at room temperature and a deep one, which releases a trapped charge only when optically stimulated. As reported before, the 275 nm excitation leads to charge trapping, but the corresponding OSL almost reached the background after 60 s of stimulation. OSL after 350 nm excitation is plotted for comparison, which does not show effective charging.

To further elucidate the optical charging pathways, Figure 3a shows the PL emission spectra of the Pr:Y_2_O_3_ sample under two different excitations. PL under 215 nm excitation exhibits two distinct emissions, a broad emission with a peak at 465 nm and a series of narrow emission lines between 610 nm and 670 nm. Under 270 nm excitation, only the narrow lines are present. These sharp lines are associated with the ^1^D_2_ → ^3^H_4_ transitions of Pr^3+^. The broadband emission likely originates from relaxed self-trapped excitons (STE) in Y_2_O_3_, which were shown to have a broadband emission centered at 359 nm (3.45 eV) with the shorter wavelength tail reaching 275–295 nm [37], [38]. The discrepancy compared to our result can be explained by the inefficiency in our setup, which is not optimized for detecting STE emission from Y_2_O_3_ (Figure S4, Supplementary Materials). As the excitation wavelength moves from 215 nm towards 270 nm, the broadband vanishes almost completely (Figure S5, Supplementary Materials), as expected from an STE emission. One might also argue that such emission is due to the presence of F-centers or F^+^-centers. Reports have shown that oxides such as Al_2_O_3_ (Sapphire), Y_3_Al_5_O_12_ (YAG) and YAlO_3_ present F-center or F^+^-center emissions with peaks ranging from 413 nm to 464 nm [32], [39]. However, in our study, no other excitation band was found to promote such broadband emission.

As depicted in Figure 3b, the PLE spectra were collected by probing the Pr^3+^ emission around 630 nm. At shorter wavelengths, a clear excitation band centered at 275 nm appears, which is due to the spin-allowed 4f-5d transitions of Pr^3+^. The relative increase of the background for excitation wavelengths longer than 340 nm is due to the relatively low output of the deuterium lamp at such wavelengths (Figure S6, Supplementary Materials).

Next, we measured the OSL emission spectra to understand the charge recombination pathways during optical stimulation. As shown in Figure 4a, two OSL emission bands are present: a broadband with a peak centered at 470 nm and narrow emission lines around 630 nm. These emission bands are identical to those PL emission bands observed under 215 nm excitation. Therefore, two radiative recombination pathways take place after 215 nm charging: one that leads to the Pr^3+^ emission and another one responsible for the self-trapped exciton emission.

Thermoluminescence (TL) curves from room temperature up to 400 °C were collected to provide information about the charge traps present in Y_2_O_3_. As shown in Figure 4b, the undoped sample exhibited a TL peak at 65 °C after 215 nm charging. On the other hand, the Pr-doped sample presented two main TL peaks, one centered at 90 °C and another one centered at 320 °C. This result explains the presence of two components observed during OSL measurements, i.e., the spontaneous emission at room temperature, which comes from a shallow trap, and the OSL itself, which arises from a deep trap when optically stimulated. The peaks at 65 °C and 90 °C are probably related to the same defect in the host. The introduction of dopants leads to distortions in the lattice. As a result, native defects and their probability of charge release with temperature changes are likely altered (represented by the temperature shift of the TL peaks). Charging under 275 nm also leads to two TL peaks, i.e., one that corresponds to a shallow trap and another one that corresponds to a deep trap (Figure S7, Supplementary Materials). But the TL response is much weaker than that after 215 nm charging, which is in agreement with the OCT results.

For the additional TL peak centered at 320 °C, that might be either introduced by a host defect during Pr doping or introduced by the dopant itself, since Pr^3+^ is known to act as a hole trapping center in some rare-earth codoped compounds [40], [41], [42]. However, more studies are needed to shed light on this, since Pr^3+^ is unlikely to act as both charge trap and recombination center (as shown to be the case in Figure 4a). The trap depth (*E*) associated to these TL peaks were estimated by the approximation *E* = *C*_*U*_ *k*_*B*_ *T*_*m*_, where *C*_*U*_ is the called Urbach constant (18 ≤ *C*_*U*_ ≤ 44), *k*_*B*_ is the Boltzmann constant and *T*_*m*_ is the temperature of the TL peak [25]. Taking the average value of *C*_*U*_ as reference (*C*_*U*_ = 31), the estimated trap depths are 1.0 eV and 1.6 eV.

Based on density function theory (DFT) calculations, a previous report has shown that the energies required to change the charge state of oxygen vacancies in Y_2_O_3_, i.e., V_o_^+/2+^ and V_o_^0/+^ are 2.7 eV and 2.9 eV above the top of valence band, respectively, although they have underestimated the Y_2_O_3_ band-gap (4.1 eV) [7]. A common method of defect energy level correction when the band-gap is underestimated is to move these levels upwards as the band gap is opened up [43]. Considering our estimation of the band-gap (5.7 eV) based on the OCT spectrum, the neutral and singly charged oxygen vacancies energy levels might move up by 1.6 eV, which put them at 1.2 eV and 1.4 eV below the bottom of conduction band. Therefore, we speculate that neutral or singly charged oxygen vacancies can be the defects responsible for the charge trapping in our measurements.

To sum up the spectroscopy results, we propose a semi-empirical model for the charging-recombination processes in Pr:Y_2_O_3_ (Figure 4c). The band-gap of Y_2_O_3_ was estimated as 5.7 eV. From photoconductivity measurements, the ground state level of Pr^3+^ in Y_2_O_3_ was reported to be at 1.7 eV above the top of valence band [44]. Two charge traps in the Pr-doped sample are present, a shallow one with trap depth of 1.0 eV and a deep one with trap depth of 1.6 eV, possibly related to neutral and singly charged oxygen vacancies. Two radiative recombination pathways during optical stimulation were found, i.e., one that leads to the narrow ^1^D_2_ → ^3^H_4_ transitions of Pr^3+^ and another assigned to a self-trapped exciton emission. When excited at 215 nm, valence electrons overcome the band-gap and are captured by the charge traps. At room temperature, the shallow traps (ST) spontaneously release the electrons with sufficient thermal energy. When optically stimulated, electrons captured by the deep traps are released and recombine both through excited states of Pr^3+^, as well as with a self-trapped hole (thus, the self-trapped exciton emission).

**Figure 5:**
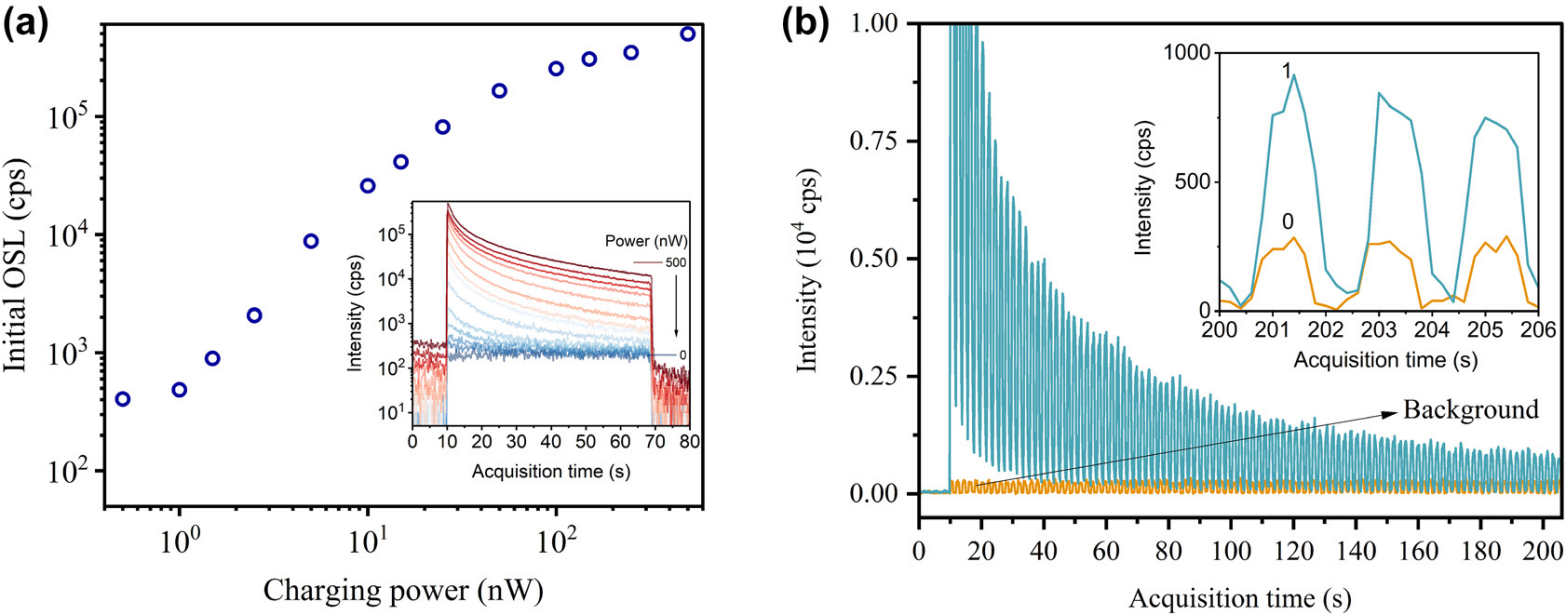
Dynamic range of the charging process and repetitive OSL readout. (a) Initial OSL intensity for Pr:Y_2_O_3_ after different excitation powers under 215 nm (60 s charging): 0, 0.5, 1, 1.5, 2.5, 5, 10, 15, 25, 50, 100, 150, 250, 500 nW. Inset shows the corresponding OSL curves. 0 nW measurement corresponds to the background. (b) 100-cycle OSL intensity readout performed after a single shot charging of a Pr:Y_2_O_3_ sample. The main figure shows all the OSL cycles following a 10 s delay after starting signal acquisition. Inset highlights a few cycles at about 200 s, where discrimination between the charged (‘1’) and uncharged (‘0’) remains clear. Each cycle has a period of 2 s and 1 s stimulation time. Charging is performed under 215 nm at 100 nW over 60 s. Background (‘0’) refers to the PMT response from the uncharged sample.

The dynamic range of the optical charging process was evaluated by measuring the OSL response after different charging intensities under 215 nm excitation, with the charging time fixed at 60 s. As shown in Figure 5a, the OSL intensity increases monotonically across the 0.5 – 500 nW power range. As the charging intensity is increased, the probability of charge trapping increases, and a finite number of traps in the host explains the saturation at high charging intensities. Increasing the density of native defects by optimizing the synthesis of these materials could extend the non-saturating power range of the OSL. Interpolating the OSL decay curve until full depletion, the total OSL photons after 1 μW charging is ∼3 × 10^6^. Considering this, we estimated the total number of stimulated OSL photons per unit volume in Y_2_O_3_ as ∼2 × 10^8^ mm^−3^, which lower bounds the density of charge trapping defects in the sample. This estimate takes into account the 1 % photon collection efficiency due to finite PMT aperture, the 47 % transmission efficiency of the filters and 5 × 10^5^ counts/pW/s sensitivity (450 nm) of the PMT.

The combination of effective charging and OSL processes allows us to control the trapped charge density in the host material on-demand. This control is not only essential for realizing charge-based optical memories but also lend itself to a potential tool for enhancing the optical coherence times of rare-earth quantum emitters in the host matrix. As a proof-of-principle demonstration, we performed repeated optical stimulation and OSL intensity readout after the Pr:Y_2_O_3_ sample is charged by 215 nm excitation, and the result is compared with the case of the uncharged sample. As depicted in Figure 5b, with intermittent stimulation at 1 s interval, the detrapped charges measured by OSL intensity decays at a characteristic time of 48 s. Here, the stimulation intensity was 5 mW/cm^2^. Treating the sample as a digital memory, we see a clear distinction between the charged (i.e., bit ‘1’) and uncharged (i.e., bit ‘0’) states even after >200 s stimulation (inset of Figure 5b). Further optimization such as reducing the stimulation intensity, increasing the charged area (7 mm^2^ in current experiment), improving the OSL collection efficiency or cooling the sample to cryogenic temperatures would lead to a slower decay, more readout cycles and further improved discrimination between the ‘0’ and ‘1’ states.

## 4 Conclusion and outlook

We have reported a versatile experimental approach, i.e., optical charge trapping spectroscopy, to control the charge state of lattice defects in a rare-earth doped oxide host crystal with only optical excitations. Excitations *via* the inter-band absorption of the host and 4f-5d transition of Pr^3+^ were found to be effective for charging the defects in the host. Recombination pathways were found to be *via* Pr^3+^ relaxation and *via* a host-related process (possibly self-trapped exciton relaxation). Besides reporting an all-optical control approach of localized charging environment, this work offers a tool to probe possible interactions between intrinsic defects and luminescence centers in the host matrix. Further studies could investigate the dynamics of charging and OSL readout processes, by varying the charging and stimulation pulse widths and the wait time between them. The physical origin of these charge trapping defects, such as tunneling two-level-systems (TLS) or F^+^ oxygen vacancy centers, also warrants further research. The optical charging spectroscopy technique can aid the development of high density optical storage systems based on atomic-scale charge trapping defects in solids without the need of high-energy (X-ray) light sources. In view of that, localized charge control can be realized by performing charging and OSL readout through a high-NA microscope objective to reach a diffraction-limited spatial resolution. Such a nanoscopic control of the local charge environment around individual rare-earth quantum emitters [45] could be used to suppress fluctuating noise and spectral diffusion, and in turn enhance the quantum coherence of single rare-earth ions for quantum information applications.

## Supplementary Material

Supplementary Material Details
