# Supplementary material for: All-optical control of charge-trapping defects in rare-earth doped oxides
Source: Nanophotonics. 2025 Feb 14;14(11):1827–35. doi: 10.1515/nanoph-2024-0635 (PMC12133311; doi:10.1515/nanoph-2024-0635)
Supplement: Supplementary file 1 — Supplementary Material Details [file j_nanoph-2024-0635_suppl_001.pdf]

Supplementary Materials

All-optical control of charge-trapping defects in rare-earth doped oxides

Leonardo V. S. França[a,*] *Shaan Doshi*[b] *Haitao Zhang*[c] *Tian Zhong*[d,**]

[a,b,d] Pritzker School of Molecular Engineering, University of Chicago, Chicago IL, United States of America
[c] Corning Research & Development Corporation, Sullivan Park, Painted Post, New York 14870, United States of America

[*] Email: leofranca@uchicago.edu
[**] Email: tzh@uchicago.edu

Figure S1 shows five OSL curves collected under the same experimental conditions for estimation of the measurement error. Same charging and OSL intensity readout setups described in the main text were used here. Bleaching was performed prior to all OSL measurements to ensure that the sample was reset.

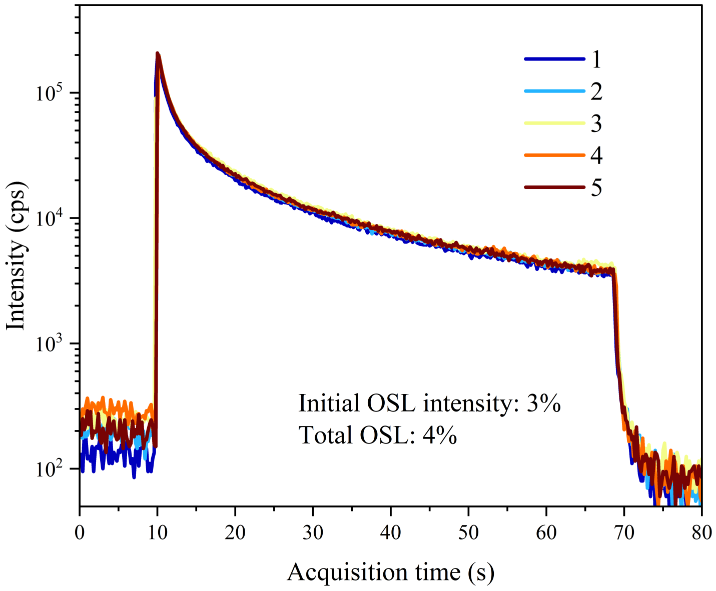

Figure S1: OSL after 215 nm charging (5 nm linewidth, 300 nW over 60 s) collected five times. OSL intensity readout was collected under CW 532 nm optical stimulation. The figure shows the standard deviations for the initial OSL intensity and the total OSL integrated over 60 s of stimulation.

Figure S2 shows the OCT spectrum of the undoped Y_2O_3 sample around the 4f-5d transition of Pr^{3+}. OCT response after 215 nm is shown for comparison. Initial OSL intensity after 215 nm charging is at least 50 times lower than the Pr-doped sample response (see Figure 2c, main text).

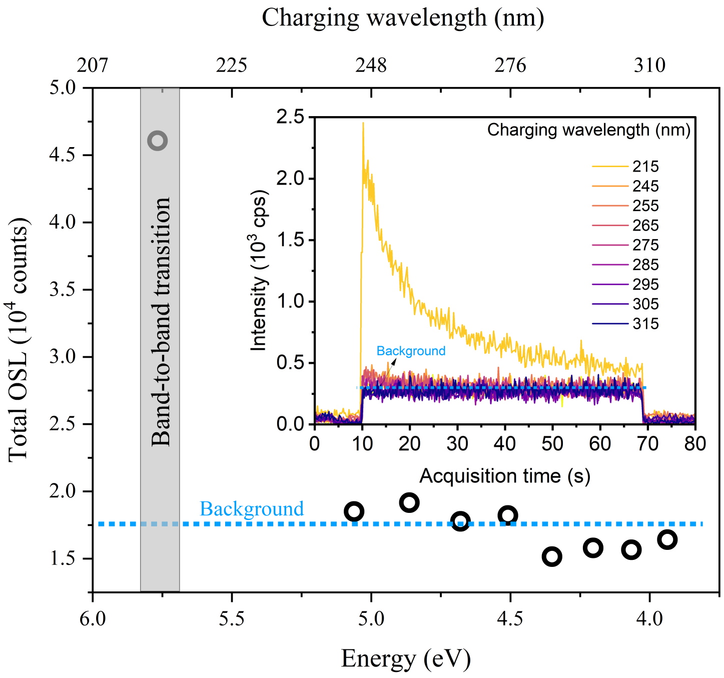

Figure S2: OCT spectrum of undoped Y_2O_3 sample. Charging parameters: 300 nW over 60 s, 5 nm linewidth and 10 nm step size. Stimulation and readout parameters were the same used with doped sample (see main text). Charging after 215 nm is shown for comparison. Inset shows the corresponding OSL curves.

Figure S3 shows the PMT response with no optical stimulation after different charging wavelengths. That was measured during the first 10 s of acquisition before optical stimulation started. Clearly, 215 nm excitation leads to spontaneous emission, which suggests that shallow traps are present in the doped sample.

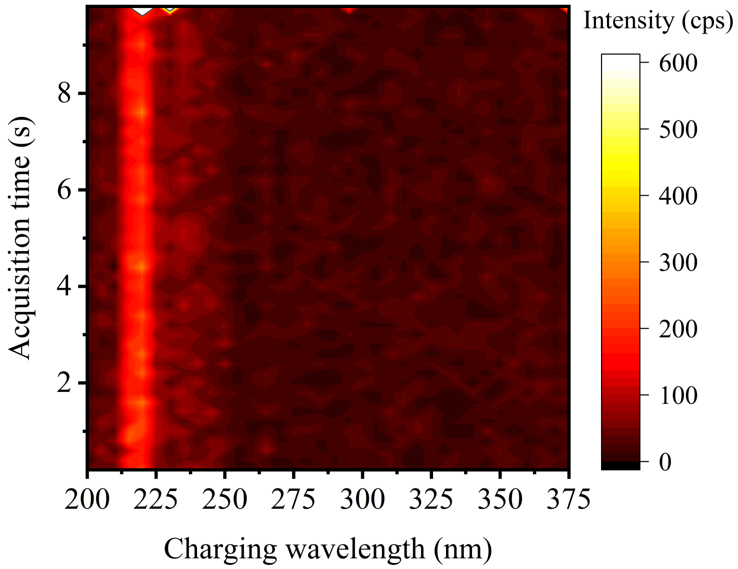

Figure S3: PMT response with no optical stimulation after different charging wavelengths.

Figure S4 shows the transmission spectrum of the collecting lens used in the PL/PLE measurements and the spectrometer responsivity. Clearly, the lens is not appropriate for UV detection such as the self-trapped exciton emission of Y_2O_3 (peak at 359 nm and tail reaching 275-295 nm). Also, the spectrometer responsivity, with grating blazed at 500 nm is not optimized for UV detection. Responsivity below 350 nm is not available.

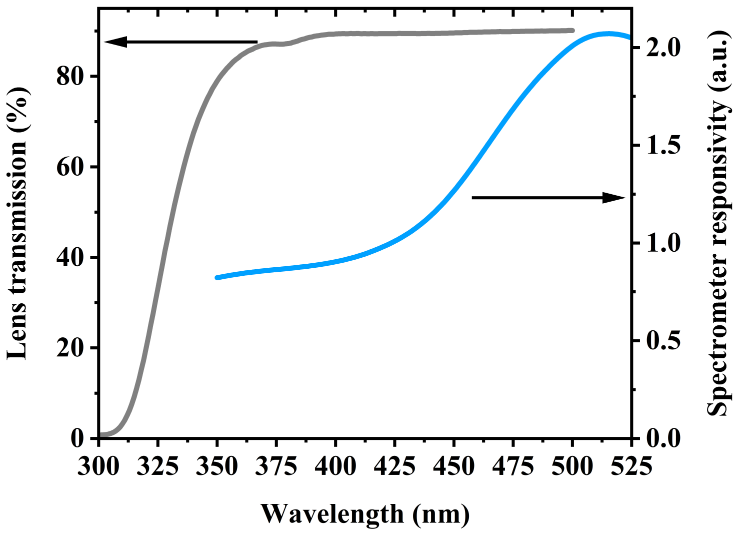

Figure S4: Transmission of aspheric lens used to collect light from the sample and spectrometer responsivity, which does not include the lens transmittance.

Figure S5 shows the PL of Pr:Y_2O_3 at different excitation wavlengths. Clearly, the broad emission vanishes when excitation moves to longer wavelengths. No other excitation bands was found. That corroborates with the interpratation that the broadband emission comes from a relaxed self-trapped exciton.

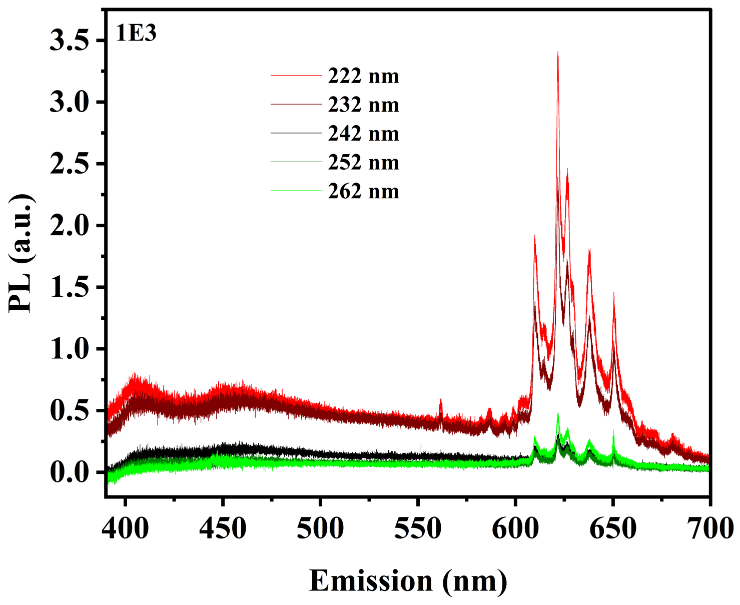

Figure S5: PL of Pr:Y_2O_3 sample under different excitation wavelengths.

Figure S6 shows the spectral irradiance of the UV lamp used in PL/PLE experiments.

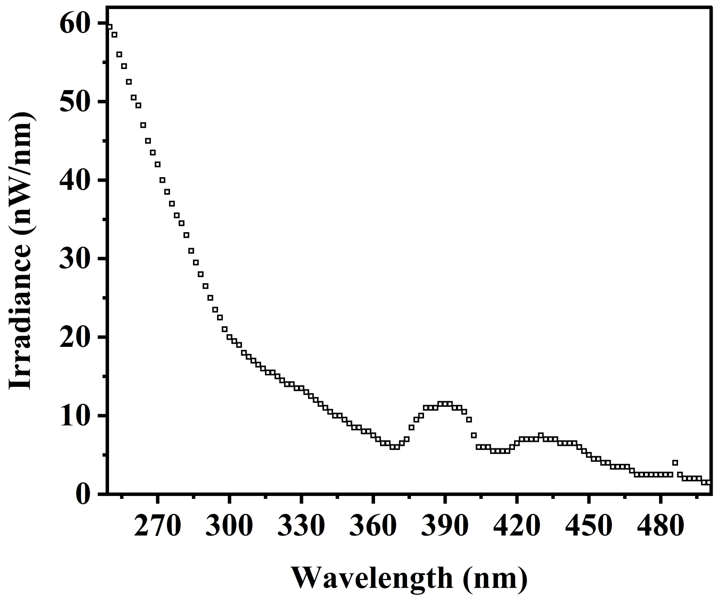

Figure S6: Spectral irradiance of deuterium lamp. Power measured with a photodiode power sensor (S120VC, Thorlabs) after UV monochromator (2 nm linewidth).

Figure S7 shows the thermoluminescence curves of Pr- doped sample after 275 nm charging. Two TL peaks centered at 95 °C and 275 °C are present.

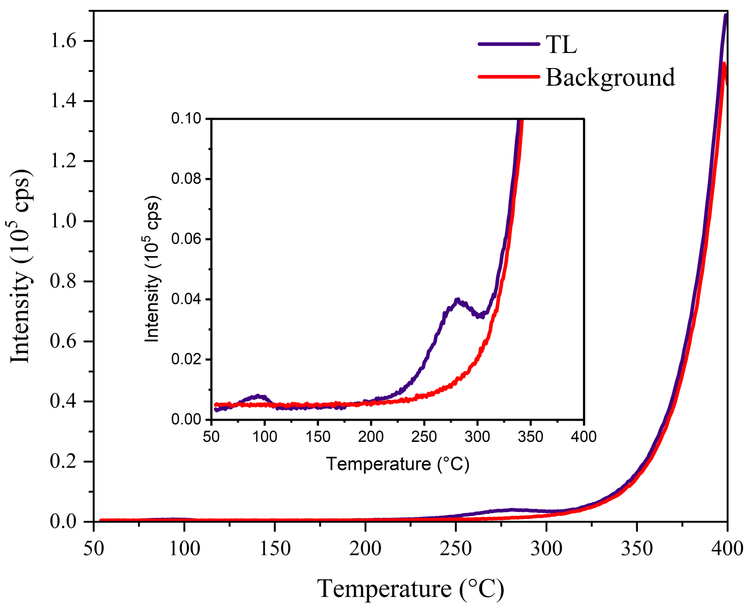

Figure S7: TL curve of Pr- doped sample after 275 nm charging (1.5 μW optical power over 60 s, 5 nm linewidth). 450 nm bandpass filter in front of the PMT was used. Background was collected right after the thermoluminescence readout. Inset shows a zoomed-in region for better visualization of TL peaks. Heating rate used: 1°C/s.
